# Stakeholder opinion on the proposal to introduce ‘treat and referral’ into the Irish emergency medical service

**DOI:** 10.1186/s12873-019-0295-5

**Published:** 2019-12-21

**Authors:** Brian Power, Gerard Bury, John Ryan

**Affiliations:** 1 Pre-Hospital Emergency Care Council, Beech House, Millennium Pk, Naas, Co., Kildare, W91 TK7N Ireland; 2 Centre for Emergency Medical Science, University College Dublin, Dublin, Ireland; 3 Emergency Department, St Vincent’s University Hospital, Dublin, Ireland

**Keywords:** Paramedic, Consultant in emergency medicine, Emergency departments, Admission avoidance, Prehospital care, Treat and referral, Treat and release

## Abstract

**Background:**

The Irish ambulance services have traditionally transported all patients following an emergency (112/999) call, regardless of acuity, to an emergency department (ED). A proposal to introduce Treat and Referral, an established care pathway in some jurisdictions, is under active consideration in Ireland. This will present a significant change. Stakeholder engagement is recognised as an essential component of management of such change. This study has conducted a multicentre, cross-sectional survey exploring opinions on the introduction of Treat and Referral among key Irish stakeholders; consultants in emergency medicine, paramedics and advanced paramedics.

**Methods:**

Public-sector consultants in emergency medicine (EM), registered paramedics and advanced paramedics, in Ireland at the time of the study, were invited to complete an on-line survey.

**Results:**

A significant finding was that 90% of both cohorts (EM consultants and registered paramedic practitioners) support written after-care instructions being given to referred patients, that > 83% agree that Treat and Referral will reduce unnecessary ambulance journeys and that 70% are in favour of their own family member being offered Treat and Referral. Consensus was reached between respondents that Treat and Referral would improve care and increase clinical judgement of practitioners. Differences were identified in relation to the increased availability of ambulances locally, that only adults should be included, and that research was required to extend Treat and Referral beyond the index conditions. There was no consensus on whether general practitioners (GPs) should be directly informed.

**Conclusions:**

This study identified that the Irish healthcare practitioners surveyed are supportive of the introduction of Treat and Referral into Ireland. It also affords healthcare policymakers the opportunity to address the concerns raised, in particular the clinical level which will be targeted for inclusion in this extended scope of practice.

## Background

The international literature has identified that a significant proportion of patients transported to an emergency department (ED) by ambulance do not have life-threatening conditions [1 2] and do not necessarily require an ambulance to get to an ED. [1–3] The Health Service Executive report a 3.7% year on year increase in ED attendances in the 2018 annual report [4]. Furthermore, pre-hospital emergency care practice has demonstrated safety and efficacy in managing some acute presentations alleviating the need for immediate ED care [5–8]. Patients, carers and bystanders perceptions of clinical urgency, resulting in ambulance use, appear to be far greater than the actual clinical problem [3–9]. The literature suggests that between 30 and 50% of patients attending ED could be appropriately treated in less emergent settings [10–13]. Indeed one author has claimed that up to 80% of these inappropriate ED attenders could be treated adequately in a primary care setting [14].

Currently, for all patients in Ireland, ambulance transport to an ED or Percutaneous Coronary Intervention centre is the only option which can be offered by paramedics and advanced paramedics. Similarly, the traditional role of paramedics in North America has been to examine, treat, and then transport patients to an ED. [15, 16] This contrasts with UK and Australian ambulance services which have transitioned to non-conveyance of selected patients [15, 17, 18].

Drivers for the introduction of non-conveyancing strategies such as ‘Treat and Refer’ or ‘Treat and Discharge’ include improving patient quality of care, maximising the utility of ambulance services, easing ED workloads or responding to patient experience [16, 19–21]. Overcrowding in EDs is an international issue and ambulance bypass is seen as a potential solution [22–24]. The introduction of Treat and Referral in the UK was associated with a substantial reduction in ambulance service conveyance rates, from 90 to 58% over a twelve-year period [18].

In Ireland, paramedics and advanced paramedics are regulated by the Pre-Hospital Emergency Care Council (PHECC). Paramedics are educated to diploma level and provide intermediate life support as part of a two-paramedic ambulance crew responding to all emergency medical incidents. Advanced paramedics (APs) receive additional education to post graduate diploma level and provide advanced life support (ALS) as solo responders or part of an ALS ambulance response. APs make up 20% of the paramedic practitioners in Ireland.

In recent years, disposition options for both clinical levels have been introduced by PHECC for ST elevation Myocardial Infarction, stroke and certain trauma presentations, permitting by-pass of the nearest ED in order to travel to a specialist centre [25]. Pre-hospital emergency care interventions have improved significantly over the decades and specific acute presentations can be definitively managed through these interventions, reducing the requirements for ongoing immediate acute care [26–29] The ability of paramedics to universally make decisions in relation to Treat and Referral, however, has not been established in the peer-reviewed literature [30]. Furthermore, the available evidence does not support practitioners below that of an Irish advanced paramedic making such decision s[31–33].

In a regional stakeholder study, to explore attitudes and perceptions of healthcare providers in relation to inappropriate attendance at the ED, Breen and McCann (2013) [34] surveyed doctors, nurses and paramedics in three hospitals in Ireland. This questioned inter alia whether; *‘Ambulance staff should have the choice whether to transport a patient to the ED or the general practitioner (GP)’*. Authorising ambulance personnel to decide on transporting the patient to a primary care facility or to an ED had significant support. While doctors and nurses were cautious, paramedics had a much greater proportion in favour; it is unclear from the research why this difference exists. Rice (2016) [35] reported, in a survey of Dublin paramedics and advanced paramedics, that a significant majority agreed that alternative care pathways to the ED was a good idea. Exploration of the views of these groups and increased understanding for their differing perspectives is required.

While Treat and Referral has been introduced in other jurisdictions for some time, there remain concerns in relation to this pathway among medical practitioners in these and other countries [36–39].

## Methods

### Design

This study engaged consultants in emergency medicine, paramedics and advanced paramedics in Ireland in relation to the proposed introduction of Treat and Referral, as stakeholder buy-in is necessary for change management success [40]. Other stakeholder cohorts, general practitioners and patients /carers were engaged in separate research exercises, which will be reported on separately. Treat and Referral was defined as the process whereby a paramedic treats a patient, following a 112/999 incident, and offers a disposition other than ambulance transport to an ED. [2–19, 38, 41]

### Participant and setting

The population consisted of consultants in emergency medicine in the public sector in Ireland, who were identified through the Irish Medical Directory [42], and paramedics and advanced paramedics on the PHECC register. The initial sample frame was defined by EM consultants and practitioners who had an e-mail address. An invitation to respond to the survey was sent through e-mail followed by reminder e-mails. A delivery receipt was requested with the e-mails sent. The final sample size was therefore determined by e-mails delivered verified by a delivery receipt.

### Instrument

On line anonymised questionnaire surveys were circulated to consultants in emergency medicine, paramedics and advanced paramedics to explore their perceptions and views of the introduction in Ireland of Treat and Referral options for patients who had recovered from hypoglycaemia or isolated seizure.

Electronic surveys were constructed using an online survey tool (Survey Monkey). Consent was received from respondents through voluntary participation in the survey. The surveys were piloted, in paper form, to assist with face validity and a number of iterations of the survey were developed to ensure appropriate wording and content [43]. Hypoglycaemia and seizure were the index presentations under consideration for Treat and Referral, as these presentations may be definitively managed in the pre-hospital environment [5–8, 44, 45].

The survey, which included a detailed description of the research, had seven domains: [46] demographics [47] experience with hypoglycaemia and seizure management [1] opinion on Treat and Referral introduction, [2] patients declining transport, [3] training / confidence in care management, [4] communication and [5] capacity assessment. The declining transport, communication and capacity assessment domains are reported on elsewhere. A combination of question types was utilised, including dichotomous, ordinal polytomous (5-point Likert scales [1 = strongly disagree to 5 = strongly agree]) and open-ended questions.

### Analysis

Data was downloaded into an Excel spreadsheet (Microsoft). The data was coded for and imported into, IBM SPSS Statistics 20 software for analysis. Cross-tabulation and frequency distribution were used to interpret the quantitative data. Median values were used to interpret the results for the Likert scales. For analysis the Likert scale was collapsed into a trichotomous scale (disagree, neutral, agree). Jeong (2016) [48] established that reliability or validity of the questionnaire is not reduced as a result of this conversion. Confidence intervals were calculated at 95% using an on-line calculator [49]. Pearson’s Chi square tests was used to identify statistically significant differences among cohorts. Statistical significance was taken at a level of *p* < 0.05.

### Ethics

Ethical approval was obtained through the University Hospital Limerick Ethics Committee. Informed consent was obtained through voluntary completion of the survey by respondents.

## Results

### Response rates

Response rates differed between the clinical cohorts, *n* = 375 paramedics (27% of paramedics who received the survey), *n* = 244 advanced paramedics (80% of advanced paramedics who received the survey) and *n* = 39 (62% of consultants in emergency medicine who received the survey).

### Demographics

Representative stakeholder opinion was achieved across case-mix, ED attendance rates and geographical spread. Table 1 summarises respondents principal work setting by urban /rural mix.

**Table 1:** Service area and clinical level of respondents

Service area	Paramedic	Advanced Paramedic	Emergency Medicine Consultant	Total
Totally urban	50	14	6	70 (10.6%)
Mainly urban	164	116	19	299 (45.5%)
Mainly rural	150	102	13	265 (40.3%)
Totally rural	11	12	1	24 (3.6%)
Total (response rate)	375 (27%)	244 (80%)	39 (62%)	658

A maximum distance of travel to ED was collapsed into two groups ≤20 Km and > 20 Km for analysis. No statistically significant difference was identified between all three cohorts of respondents in relation to service area and travel time to ED. This also applied to the opinions on treat and referral Table 2.

**Table 2:** Geographical spread of EM Consultant respondents

Area	Respondents	Percentage per area
Dublin City	12	41.4%
Mid Leinster area	5	100.0%
North Eastern area	3	75.0%
Southern area	10	66.7%
Western area	9	64.3%
Total	39	58.2%

The majority of consultants (95%) report an ED attendance of > 30,000 per annum while the balance (5%) report attendance of 20,000–30,000 at their ED.

### Survey answers

Respondents’ opinion on 11 statements in relation to Treat and Referral was sought. Table 3 summarises these responses. There was strong agreement on key statements. Particularly, that ‘Treat and Referral will result in improved patient care’. A significant finding was that 90% of both cohorts (EM consultants and PHECC practitioners) support written after-care instructions being given to referred patients, that > 83% agree that Treat and Referral will reduce unnecessary ambulance journeys and that 70% are in favour of their own family member being offered Treat and Referral. The scale for these statements had a good level of internal consistency as determined by a Cronbach’s alpha of 0.671.

**Table 3:** Survey statements on Treat and Referral

Text	Median score (range)	PHECC practitioner agree/ strongly agree (CI 95%)	EM Consultant Agree/ strongly agree (CI 95%)	Statistical difference between PHECC practitioners and EM Consultants
T&R will result in improved patient care.	4 (1–5)	66.5% (±3.5%)	61.1% (±10.2%)	*p* = 0.346
T&R will increase clinical judgement skills.	4 (1–5)	73.5% (±3.5%)	61.1% (±10.2%)	*p* = 0.023
T&R will reduce unnecessary ambulance journeys.	4 (1–5)	87% (±3.5%)	83.3% (±10.2%)	*p* = 0.053
T&R will result in increased ambulance availabilities for emergencies locally.	4 (1–5)	83.9% (±3.5%)	55.6% (±10.2%)	p < 0.001
T&R should only be available as an advanced paramedic intervention.	2 (1–5)	22.6% (±3.5%)	57.2% (±10.2%)	*p* = 0.001
T&R should only be available as an intervention to paramedics with several years’ experience.	3 (1–5)	45.4% (±3.5%)	47.2% (±10.2%)	p = 0.525
T&R should only be available for adult patients (18 and over).	4 (1–5)	57.2% (±3.5%)	47.2% (±10.2%)	p = 0.005
I would be happy for a family member to be offered T&R by a paramedic or advanced paramedic following an acute event.	4 (1–5)	69.6% (±3.5%)	69.4% (±10.2%)	*p* = 0.567
Patients offered T&R should be given specific written after-care instruction, similar to head injury advice leaflet given by emergency department staff.	4 (1–5)	88.2% (±3.5%)	91.7% (±10.2%)	*p* = 0.744
Patients offered T&R should be limited to specific conditions such as hypoglycaemia and isolated seizure until research demonstrates it is a safe clinical practice.	4 (1–5)	50.6% (±3.5%)	69.4% (±10.2%)	p = 0.013
Patients offered Treat and Referral will require their GP to be informed about the episode through e-mail or ordinary mail by the treating paramedic or advanced paramedic.	3 (1–5)	47.5% (±3.5%)	88.9% (±10.2%)	p < 0.001
Mean	3.6	62.9%	66.6%	

‘Limiting Treat and Referral to hypoglycaemia and seizure until research demonstrates it is safe to do so’ had a slight majority of PHECC practitioners (51%) in agreement and a majority of EM consultants (69%) in agreement. The PHECC practitioners’ opinion was statistically significantly different to that of the EM consultants (*p* = 0.013).

‘Treat and Referral will increase ambulance availability for emergencies locally’ had a significant majority of PHECC practitioners (84%) in agreement but only a small majority of EM consultants (56%) were in agreement. There was a highly statistically significant different between PHECC practitioners and EM consultants on this statement (*p* < 0.001).

The clinical level at which PHECC practitioners should have Treat and Referral within their scope of practice has a difference of opinion. ‘That Treat and Referral should only be available for advanced paramedics’ was strongly opposed by the paramedic cohort (*n* = 249, 70%), while supported by a minority of advanced paramedics (*n* = 91, 39%) and a small majority of EM consultants (*n* = 20, 57%). There is a highly statistical difference between PHECC practitioners and EM consultants on this statement (*p* < 0.001).

‘That Treat and Referral should only be available for paramedics with several years-experience’ was supported by a minority of paramedic respondents (*n* = 144, 41%), a small majority of advanced paramedic respondents (*n* = 124, 53%) and a minority of EM consultant respondents (*n* = 17, 47%). There is no statistical difference between PHECC practitioners and EM consultants on this statement (*p* = 0.525). These two statements demonstrate divergence of opinion on what clinical level is appropriate for Treat and Referral to be included in the scope of practice. There is a higher mean support for paramedics with experience than advanced paramedics only (47% V 36%) across the three cohorts, however paramedic respondents may have an expected personal bias, expressing a view to not restrict their scope of practice. No strong majority exists among EM consultants in favour of experienced practitioners.

‘Limiting Treat and Referral to adult patients’ was supported by PHECC practitioners (57%) whereas a minority of EM consultant respondents supported this restriction (47.2%). There is a statistical difference between PHECC practitioners and EM consultants on this statement (*p* = 0.005). This finding among PHECC practitioners was anticipated as research suggests that providing emergency care for paediatric patients can evoke anxiety and discomfort among pre-hospital practitioners [50].

‘GPs shall be informed through e-mail following Treat and Referral for their patient’ has little support from PHECC practitioners (48%) while EM consultants supported it by a large majority (89%). There is a highly statistical difference between PHECC practitioners and EM consultants on this statement (*p* < 0.001).

Finally, the mean score agreeing or strongly agreeing across all 11 statements was 67% for EM consultants and 63% for PHECC practitioners and the collective median was 3.6 from a 5-point Likert scale. This demonstrates a majority are in support of the 11 statements which suggest that they are in favour of the introduction of treat and referral.

## Discussion

In this study the healthcare stakeholders, EM consultants and PHECC practitioners, have been surveyed to elicit their opinion in relation to the introduction of Treat and Referral into Ireland. The results demonstrate that the majority view from all concerned was in favour of this proposal. This confirms and expands the findings of regional based Irish studies [34, 35]. However, it is noteworthy that differences of opinion, between and among healthcare practitioners, were identified across several areas of the survey.

Improvements in patient care following the introduction of Treat and Referral has to be measured through clinical audit incorporating structure, process and/or outcome measures [51]. Treat and referral effectiveness is currently measured in the literature by ‘repeat episodes within 72 hours’ [52–55] and ‘patient satisfaction’ [56, 57]. Whereas, a large percentage of consultants in emergency medicine and PHECC practitioners expressed the view that Treat and Referral will improve patient care, clinical audit will be required to demonstrate any improved care.

While Emergency Medical Technicians have been used successfully in research for Treat and Referral [55], concern was raised in this study about the clinical acumen of some PHECC practitioners (paramedics compared to advanced paramedics) to select appropriate patients for a Treat and Referral clinical care pathway. This was also identified in the literature where decision making in relation to non-conveyance was reported as being more difficult for lower clinical levels [9]. The clinical level at which Treat and Referral will be introduced, has identified divergences of opinion in this research. The majority agree that it should not be restricted to advanced paramedics only, however a majority of EM consultants agree that it should be while concern is evident among paramedics at limiting their scope of practice. As with any new process this study would suggest prudence in the implementation of Treat and Referral, commencing with the higher clinical level of advanced paramedic initially.

The literature is silent on whether the GP should be informed following Treat and Referral being offered to their patients. A highly significant difference of opinion exists between PHECC practitioners and EM consultants in relation to this issue (*p* < 0.001). It would appear prudent for GPs to be informed about an acute event following a Treat and Referral disposition to ensure continuity of ongoing health management. Consensus was reached in relating to offering Treat and Referral to an own family member where the majority were in favour. This suggests personal confidence in the process.

The support for an evidence-based model was demonstrated in that the majority were in favour of restricting Treat and Referral to the index presentations, hypoglycaemia and seizure, until research demonstrated the safety and efficacy of introducing other clinical conditions. Support for evidence-based medicine was progressively more strongly supported by the higher clinical levels. This may be reflective of the low level of exposure to research education in paramedic training programmes [58] and provides an opportunity for intervention. Adults only being offered Treat and Referral is consistent with other findings [50].

### Study limitations

A low response rate (27%) was noted among paramedics, which contrasts with the high response rates on the other groups. This may reflect a paramedic view of limited relevance to their role or other reasons for non-engagement. No conclusions can therefore be drawn from this study about the representativeness of paramedic views described here. Nonresponse bias was an issue as ~ 50% of delivered e-mails were not opened, verified by no read receipt received.

The study instruments have not been validated elsewhere. The limitations of anonymous electronic surveys may preclude the identification of other barriers or facilitators among respondents.

The study focused on clinical stakeholders directly involved in the provision of emergency care. However, other health care professionals, who may be requested to accept referrals, such as GPs and diabetes and epilepsy specialists were not consulted in this study.

## Conclusion

This stakeholder engagement identified that the healthcare practitioners surveyed are, in the main, supportive of the introduction of Treat and Referral into Ireland. It also provides an opportunity to address minority concerns by healthcare policy makers, in particular the clinical level which will be targeted for inclusion in this extended scope of practice.

There appears to be no appetite for paediatric inclusion in Treat and Referral at this time. Ongoing clinical audit will be essential to evidence patient safety.

In introducing Treat and Referral into Ireland, defining appropriate structure, process and outcome measurements will ensure the confidence of healthcare policy makers in entrusting PHECC practitioners to safely implement it. The support, identified through this survey, should be harnessed to assist this process and a smooth implementation of this practice, wholly consistent with the central tenant of near patient treatment in current Irish health care Policy ‘Sláintecare’ [59].

## Data Availability

The datasets during and/or analysed during the current study available from the corresponding author on reasonable request.

## Abbreviations

ED: Emergency Department
GP: General Practitioner
PCI: Percutaneous Coronary Intervention
PHECC: Pre-Hospital Emergency Care Council
T&R: Treat and Referral
UK: United Kingdom
